# The deep past in the virtual present: developing an interdisciplinary approach towards understanding the psychological foundations of palaeolithic cave art

**DOI:** 10.1038/s41598-023-46320-8

**Published:** 2023-11-03

**Authors:** Izzy Wisher, Paul Pettitt, Robert Kentridge

**Affiliations:** 1 Department of Linguistics, Cognitive Science and Semiotics, Aarhus University, Aarhus, Denmark; 2 Department of Archaeology and Heritage Studies, Aarhus University, Aarhus, Denmark; 3 Department of Archaeology, Durham University, Durham, UK; 4 Department of Psychology, Durham University, Durham, UK

**Keywords:** Human behaviour, Archaeology

## Abstract

Virtual Reality (VR) has vast potential for developing systematic, interdisciplinary studies to understand ephemeral behaviours in the archaeological record, such as the emergence and development of visual culture. Upper Palaeolithic cave art forms the most robust record for investigating this and the methods of its production, themes, and temporal and spatial changes have been researched extensively, but without consensus over its functions or meanings. More compelling arguments draw from visual psychology and posit that the immersive, dark conditions of caves elicited particular psychological responses, resulting in the perception—and depiction—of animals on suggestive features of cave walls. Our research developed and piloted a novel VR experiment that allowed participants to perceive 3D models of cave walls, with the Palaeolithic art digitally removed, from El Castillo cave (Cantabria, Spain). Results indicate that modern participants’ visual attention corresponded to the same topographic features of cave walls utilised by Palaeolithic artists, and that they perceived such features as resembling animals. Although preliminary, our results support the hypothesis that pareidolia—a product of our cognitive evolution—was a key mechanism in Palaeolithic art making, and demonstrates the potential of interdisciplinary VR research for understanding the evolution of art, and demonstrate the potential efficacy of the methodology.

## Introduction

Visual culture is universal among contemporary human societies. It has a deep antiquity, emerging in *Homo sapiens* by 100,000 years ago in the form of abstract, geometric engravings^1–3^ and was present among Neanderthals over a broadly similar timeframe^4^. The ability to produce figurative representations, however—drawings, paintings, engravings, and sculpted figurines—only appeared at the start of the Upper Palaeolithic (~ 40,000–13,000 cal BP), on current evidence produced only by *Homo sapiens,* and was overwhelmingly dominated by prey animals^5–9^. Animal themes persisted throughout the ~ 25,000 years of the Upper Palaeolithic to the near exclusion of other themes (humans, plants, landscapes) despite considerable change of thematic and stylistic conventions. It is clear that humans evolved as visually-centred animals^10^, but key questions remain about *why* human visual culture emerged in the way that it did.

Significant attempts have been made in archaeology to address these questions. In particular, the Upper Palaeolithic cave art—defined here as non-figurative and figurative motifs painted, drawn, sculpted or engraved on cave wall surfaces—record of France and Spain has been subject to extensive research, from the establishment of broad diachronic and regional styles^11,12^, production techniques and pigment use^13,14^, to broader interpretations of the art’s ‘function’^15–17^, its possible symbolic meaning^18,19^ and speculation about alternate psychological states that stimulated art production^20–23^. In recent years, archaeologists have abandoned these “umbrella” theories, favouring more systematic and contextually-sensitive attempts to understand Palaeolithic art. We are not concerned here with the semantics of the term ‘art’: discussion of this has been extensive in palaeoanthropology (see for example,^24,25^), but for our purposes we use the terms ‘visual culture, ‘art’ and ‘image(ery)’ interchangeably to refer to artificially made marks carrying meaning to their viewers. Of particular pertinence to challenging “umbrella” theories has been the critical engagement within archaeology of the term “art”^5^, through appreciating and incorporating cross-cultural perspectives of “art” beyond Western connotations^26^ and emphasising that art is inherently sensitive to context, and thus cannot be explained by one theoretical perspective. Similarly, there is rich potential for understanding the psychological nature of prehistoric art^27^. Hodgson’s^28–31^ work on the particular psychological mechanisms (e.g., pareidolia) that may have informed its emergence and development has also been influential. Here, the challenge is to generate interdisciplinary approaches that produce specific hypotheses which can be tested against the archaeological record using visual psychological methods, facilitating more nuanced interpretations of Palaeolithic art^32^. Previous interdisciplinary collaborations between archaeologists and psychologists have successfully used modern participants to inform about aspects of past human behaviours e.g., perception of engraved marks^33,34^ or cognitive requirements for stone tool production^35^. Many discussions about the psychological underpinnings of Palaeolithic art have, however, stagnated as the fragmentary nature of archaeological evidence is often insufficient for testing hypotheses when taken alone.

We believe that VR has vast potential to overcome this limitation. It allows for the construction of immersive yet controlled environments within which we can measure participants’ natural responses to presented stimuli. Virtual environments have been demonstrated to encourage a sense of presence or embodiment by participants as a result of the active engagement with stimuli in VR, particularly when participants can freely move within the environment^36,37^. This, in turn, stimulates naturalistic responses by participants, enriching the ecological validity of data that can be obtained through experimentation^38–41^. In psychology, VR has thus been increasingly adopted within both clinical^42–46^ and research contexts^47–51^. In archaeology, its use has primarily been restricted to museum contexts, for example immersive visitor VR experiences of reconstructed historic buildings or landscapes^52–54^. Only now is VR being deployed as an interpretive tool in archaeological research to facilitate the examination of fragile archaeological sites^55^, simulate lighting conditions for Palaeolithic art^56,57^, or evaluate areas of visual interest within historical buildings^58^. VR can thus integrate both psychological research methods and contextual information from the archaeological record to generate meaningful, and testable, insights into aspects of the earliest artistic behaviours. Since visual psychological effects, like pareidolia, are stimulated by holistic responses to environments (light conditions, dimensionality and materiality of stimuli), the immersive conditions of VR environments are also more suitable than traditional psychological methods for understanding how these contextual dimensions of caves may have triggered certain visual and perceptual responses.

We present here results of a pilot methodology for using VR psychology experiments in archaeological research. We developed three key hypotheses to evaluate the extent pareidolia—the perceptual phenomenon of perceiving meaningful forms in random patterns, i.e., faces in clouds—may have played a role in informing the theme, placement, and form of animal depictions in El Castillo cave (Cantabria, Spain):

### Hypothesis 1

As predicted by archaeological literature concerning the role of pareidolia in cave art making^29–32^, when viewing cave walls with the art digitally removed, participants will have pareidolic responses to the natural topographic features (concavities, convexities, ridges, cracks) of cave walls;

### Hypothesis 2

Participants’ tracked eye movements will correspond to the same features of cave walls selected by Palaeolithic artists for integration into figurative depictions. Eye tracking has been successfully used in psychological research on pareidolia to identify the features triggering pareidolic responses^59^ and more generally is frequently used to inform about what features are visually salient to participants^60^;

### Hypothesis 3

The pareidolic imagery perceived by participants will correspond to the forms depicted by Palaeolithic artists, indicating that pareidolia not only influenced placement, but also the form of Palaeolithic depictions.

To test these hypotheses and demonstrate proof of concept for this methodology, we produced immersive VR cave environments in *Unity*, a free gaming development software, that integrated 3D photogrammetric models of four real cave walls bearing Palaeolithic art. The selected walls were representative of different styles (i.e., incomplete outlines of animals through to detailed animal depictions that represented behaviour, movement, or coat colour) and techniques (i.e., painting, drawing, engraving) of art production from El Castillo that date between ~ 35,000 and 15,000 BP^61–63^. The models were first manipulated to digitally remove the art, leaving only the natural topography and colour. Fourteen participants were recruited and primed to identify Pleistocene animals, by presenting images of animals with increasing levels of discrimination difficulty. As Upper Palaeolithic people would have been visually attuned to recognising animals during hunting but the participants were not, this stage was necessary to mitigate some issues with using modern participants to inform about Palaeolithic behaviours. Participants then entered VR environments, and were provided with a handheld “torch” that replicated the intensity and movement of light cast from Palaeolithic light technologies^10,64,65^. Participants were directed to target walls, and initially instructed to observe the wall before being asked a series of questions (*Is the wall suitable to draw on? What would you draw? Why?*). Eye tracking, using the inbuilt eye tracking of the HTC Vive with a modified calibration code to reduce error (+ / − 2 degrees), allowed us to record areas of visual attention. Screen-captured videos of the VR sessions with synchronised recorded audio responses facilitated insights into the participants’ subjective experience of cave walls, particularly if they perceived natural features as evocative of animal forms. By using this novel, interdisciplinary methodology, our research provides concrete insights into the extent to which pareidolia influenced the creation of Palaeolithic figurative art in El Castillo cave.

## Results

VR allowed participants to experience immersive conditions, providing detailed insights into their perceptual responses to cave walls illuminated by a mobile, flickering light source. As the results, described below, showed significant variation in responses within and between participants, it is useful to evaluate these by panel.

### Panel EC1

**Figure 1:**
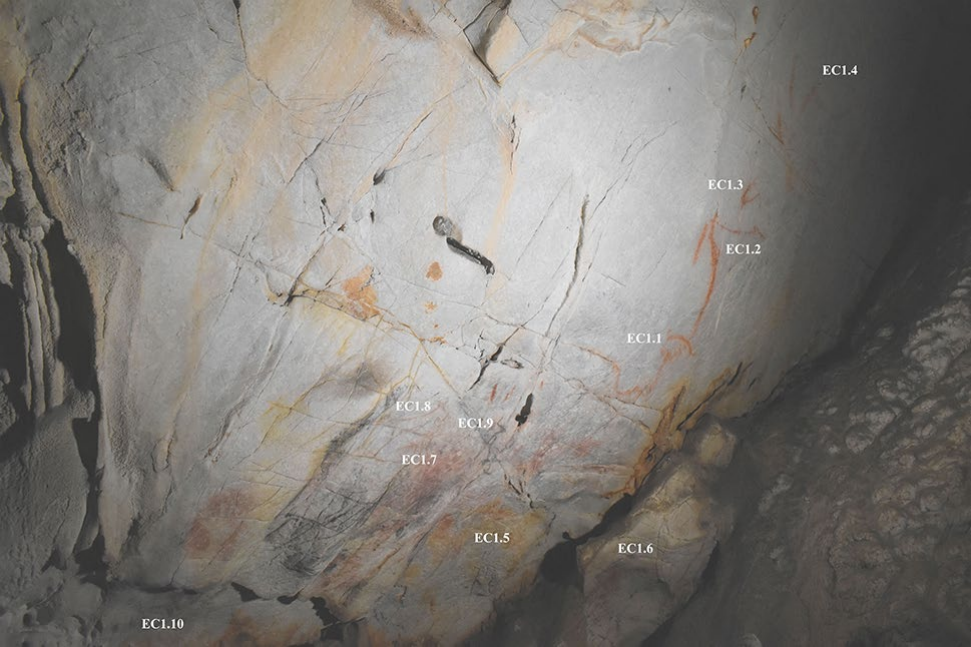
Image of Panel EC1 with individual figurative depictions labelled. EC1.1: large bison head in red; EC1.2: partial deer; EC1.3: partial horse head; EC1.4: large horse in red, EC1.5: large bison outline facing right, EC1.6: bison facing left, depicted in black outline with partial red-brown infill, EC1.7: large black bison with legs curled under body, EC1.8: hind facing right in red, EC1.9: hind facing right, originally in red but redrawn in black, EC1.10: small bison hidden under rock surface.

Panel EC1 (Fig. 1) depicts 10 Palaeolithic animals, 5 of which integrate topographic features. It elicited several different responses from the 13 participants that viewed the panel. Most participants (n = 7) appeared to experience pareidolic responses to the undecorated version of this panel, six of these perceiving evocative animal forms in the same areas used for Palaeolithic depictions (Table 1).

**Table 1:** Summary of participant responses to Panel EC1, with responses evaluated in relation to the three hypotheses.

Participant	Pareidolic response? **(H1)**	Location correct? **(H2)**	Species correct? **(H3)**	Additional comments
P1	Yes	No	No	Perceived a horse or ibex towards upper left area of the panel (same area as EC1.7)
P2	No	Yes	Yes	No apparent pareidolic response, but noted they would depict a bison and/or horse in the same area as EC1.6
P3	Yes	Yes	Yes	Perceived bison in lower, central area used for EC1.5
P4	Yes	Yes	No	Perceived horse head in natural cracks (same area as EC1.7)
P5	No	Partially	No	Used central lower section for a stag depiction, but placed depictions of deer in locations unused by Palaeolithic artists
P6	No	Partially	No	Partially used central lower section to depict a rhinoceros, (same area as EC1.5)
P7	No	Partially	No	Described different areas as suitable to depict on, broadly corresponding to those used by Palaeolithic artists. However, discusses depicting herds of animals (no specific species mentioned)
P8	Yes	No	No	Perceived fish in natural features of the wall, but did not correspond to areas used for Palaeolithic art
EP9	Yes	Yes	Yes	Perceived lower central area as evocative of a bison (same area as EC1.5)
P10	Yes	Yes	Yes	Perceived lower central areas as evocative of a bison (same area as EC1.5) and small boulder feature as evocative of a horse/bison (same area as EC1.6)
P11	No	No	No	Described depicting a deer or ibex, but not corresponding to areas used for Palaeolithic art
P13	Yes	Yes	Yes	Perceived lower central area as evocative of a bison (same area as EC1.5); also described other representations (bear, a skull) not corresponding to areas used for Palaeolithic art
EP14	Yes	Yes	No	Perceived small rock used for EC1.6 as evocative of a horse; perceived other horses in the panel which do not correspond to areas used for Palaeolithic art

Note that participant P12 had an incomplete session, and thus there are no results from P12 for this panel.

**Figure 2:**
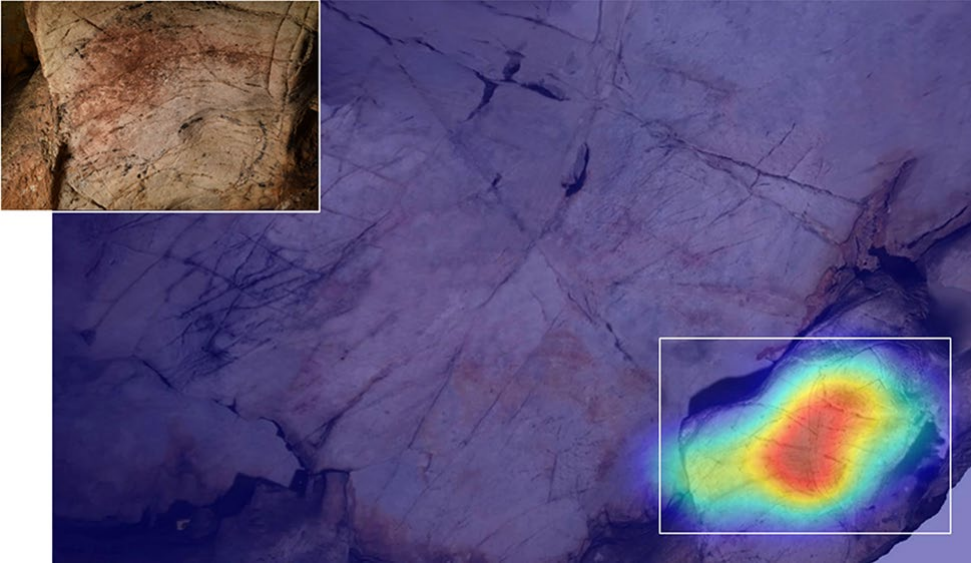
Heatmap of 25 s of tracked eye movements for P10 during active observation. The heatmap corresponds to the exact period during which P10 describes depicting a horse or bison (see quote in main text), corresponding to the same area as EC1.5.

Responses predominantly concerned three areas of the panel. The first corresponded to the small boulder slightly to the panel’s front, which had been used by Palaeolithic artists to depict a bison facing left (EC1.6). Two participants, P10 and EP14, paid particularly close visual attention to this feature, describing its natural shape as evocative of a horse or bison. For example, P10 focused their visual attention on this feature for some time whilst describing how they would depict a horse. Their audio response indicated their perceptual experience, with P10 stating “I’d use this rock as kind of a horse shape….because you’ve got the bum, and then the head or the neck. Or bison. I’d probably use that because it’s already shaped like an animal ” (Fig. 2).

Visual attention was also drawn to the lower central section, used by Palaeolithic artists to depict a black outline of a bison facing right (EC1.6). Four participants explicitly thought this area was evocative of a bison, noting that aspects of the subtle undulations and natural staining of the cave wall were bison-like in form, or that they would utilise these features to depict a bison. Two participants (P3 and EP9) also explicitly remarked that the undulations were evocative of a bison’s muscular features, after they had repeatedly moved their light source to enhance the topography of this area. P3 stated the topography was suggestive of “its big muscular forearms” and EP9 noted the topography “certainly could be used for… a yeah the back, and then draw a very muscular bison”.

**Figure 3:**
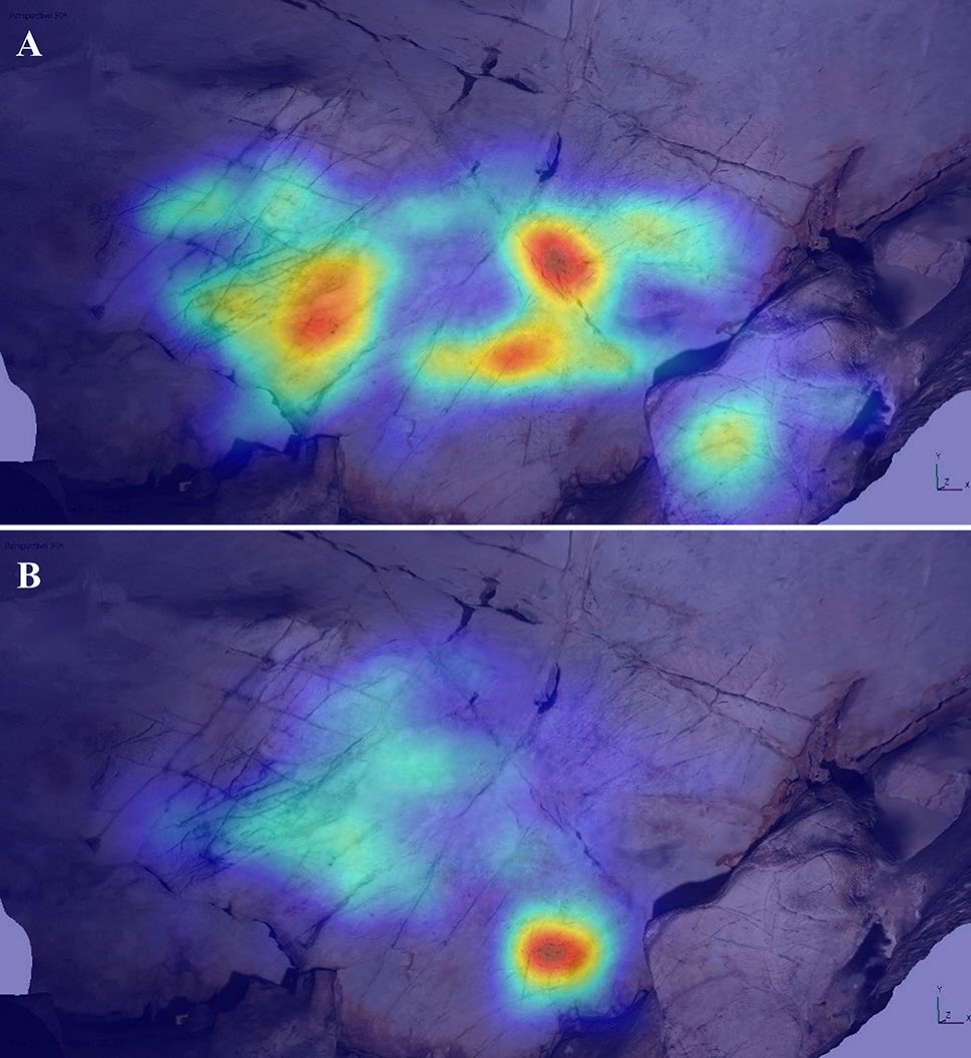
Heatmap of tracked eye movements for P10 (A) and P11 (B) during idle observation (60 s of recorded eye movements per participant). Despite both participant’s visual attention focusing on the left area, corresponding to the same area used for depiction EC1.7, neither participant had a pareidolic response to this region.

The third key area corresponded to the upper left region of the wall, utilised for a bison in a “sleeping” position, with its legs curled (EC1.7). This area received attention during the idle observation period of several participants (e.g., P10 and P11: Fig. 3), but only one (P4) appeared to have a pareidolic response, perceiving a horse head in the natural cracks utilised for the head of EC1.7. The focus of participants’ visual attention on this region may be due to the high frequency of natural deep fissures and cracks; this perhaps increased the visual saliency of this area, attracting attention but not necessarily triggering pareidolic responses.

The results from Panel EC1 thus lend some support for the three hypotheses: 7 participants perceived its features as evocative of animals (H1); 9 participants’ visual attention corresponded to the same areas used by Palaeolithic artists (H2); and, although less strongly supported, 4 participants also perceived the same animal taxa in these areas (H3). The high concentration of images on this panel perhaps increased the likelihood that participants’ responses corresponded to the same areas used by Palaeolithic artists. It is thus important that those panels with fewer depictions (EC2, EC3, and EC4) also be evaluated for the three hypotheses, to more robustly evaluate whether corresponding responses are determined by pareidolic responses to salient natural features.

### Panel EC2

**Figure 4:**
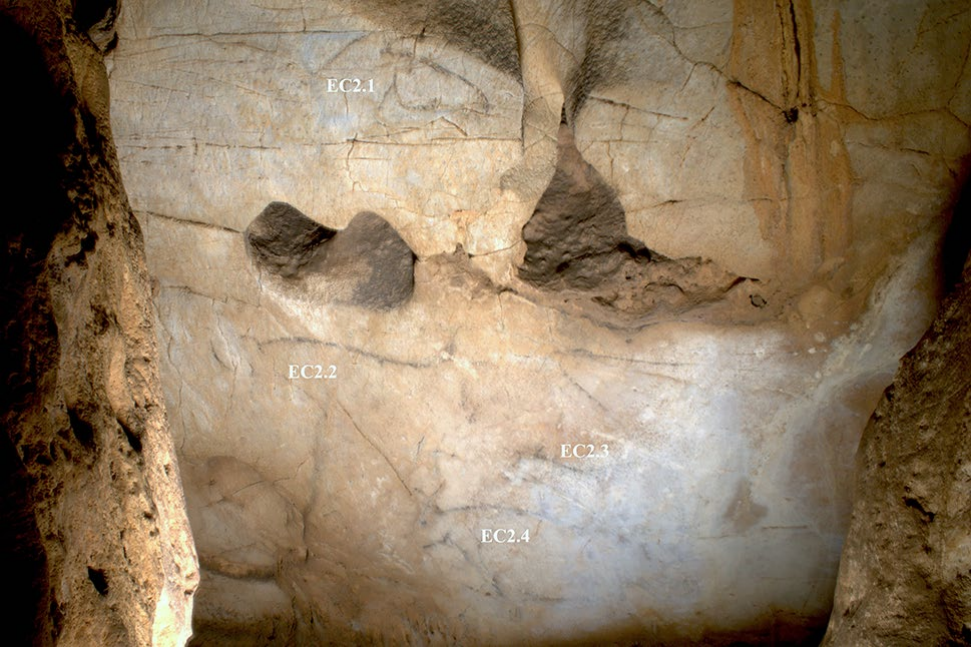
Image of Panel EC2, featuring four animal depictions. EC2.1: outline of a horse head in black; EC2.2: dorsal line of an aurochs; EC2.3: partial outline depiction of a deer facing left; EC2.4: partial outline depiction of an aurochs facing left.

**Figure 5:**
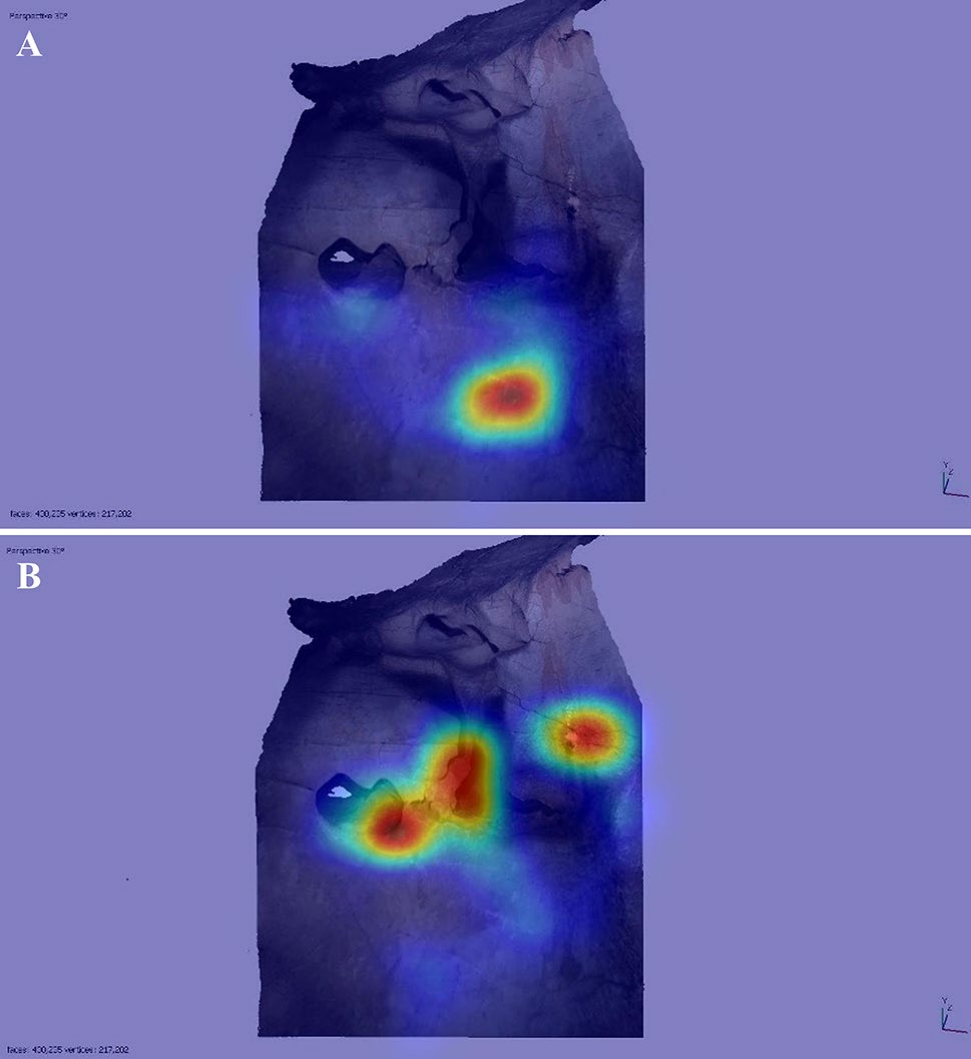
Heatmap of tracked eye movements during idle observation of Panel EC2 of P8 and EP14. (**A**) P8, who did not report pareidolia. (**B**) EP14, who did report pareidolia. There is a clear contrast in their visual attention, with EP14 focusing their attention on the undulating surface towards the upper area of the panel.

This features four simplified outline animals (Fig. 4) and elicited few pareidolic responses among participants (Table 2). Attention was primarily focused on the lower, amorphous region of the cave wall. The lack of undulating features that might obscure depictions was noted by several participants: P7 stated that the wall would be suitable to draw on “because it’s kind of large and flat and quite easy to draw on”; P8 similarly stated “it’s nice and flat so would be easy to draw a picture”; and P11 commented that “it’s a very nice flat surface”. The focus on the flatter, lower region may conform to a “blank canvas” ideal and account for participants’ not perceiving natural features as evocative of animals. For the participants that did experience pareidolia, their visual attention was focused on the upper, undulating area (Fig. 5). EP14 and P10 described pareidolic imagery of horse and/or deer within this topography, but not always corresponding to the same areas used for Palaeolithic depictions. However, EP9 did perceive the upper left area of the panel, used for a horse head depiction (EC2.1), as evocative of either a horse or bison. P11 similarly perceived an edge as evocative of the back of a bison depiction, corresponding to the same area as depiction EC2.2 of a schematised aurochs/bison.

**Table 2:** Summary of participant responses to Panel EC2, with responses evaluated in relation to the three hypotheses.

Participant	Pareidolic response? **(H1)**	Location correct? **(H2)**	Species correct? **(H3)**	Additional comments
P1	No	No	No	Used lower area of panel to represent mammoths
P2	No	Yes	No	Described using lower right area of wall (same area as EC2.3 and EC2.4) to depict ibex and chamois
P3	No	No	No	Described using lower area of the wall to depict a large mammoth or multiple horses/deer
P4	No	No	No	Described several animals (bison, horse, and mammoth) but did not correspond to areas used for Palaeolithic art
P5	No	No	No	Used lower left section of panel to draw mammoth and elk
P6	No	No	No	Used lower section of panel to depict deer or mammoth
P7	No	No	No	Described depicting mammoth on lower section of panel
P8	Yes	Yes	No	Perceived a bison in natural colour/texture of rock in lower right area (same area as EC2.3 and EC2.4)
EP9	Yes	Yes	Partially	Perceived a horse head in upper left area (corresponding to EC2.1), but changed response to depict a bison
P10	No	No	No	Described natural undulations in upper right area as looking like antelope, but did not correspond to areas used for Palaeolithic art
P11	Yes	Partially	No	Described depicting a large mammoth on lower area of panel, then identified upper edge as evocative of a bison (same area as EC2.2)
P12	No	No	No	Described depicting a large animal on lower section of the panel
P13	No	No	No	Described using lower section to depict mammoth or deer herd
EP14	Yes	No	No	Perceived multiple animals (bison, reindeer, horse) in upper area, but did not correspond to the areas used for Palaeolithic art

Participants’ responses to Panel EC2 thus contrast with those from Panel EC1 and do not appear to support the hypotheses. Only 4 participants experienced pareidolic responses and/or paid visual attention to the same areas used by Palaeolithic artists, and no participants described depicting the same animal form on the panel as those depicted in the Palaeolithic. For this particular panel, therefore, it appears that the production of the depictions may not have been strongly motivated by the natural features of the cave wall.

### Panel EC3

**Figure 6:**
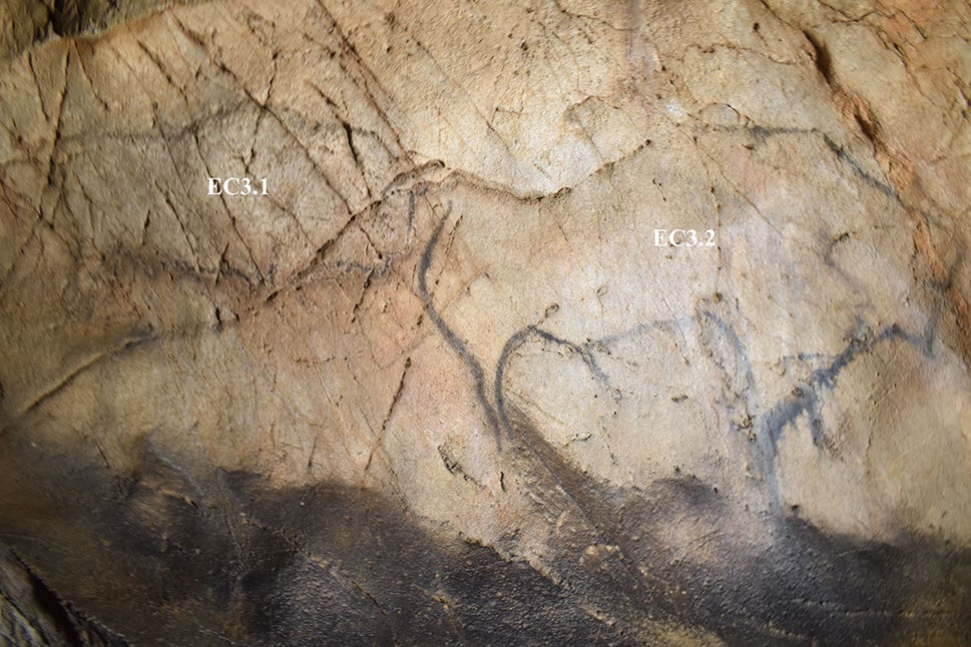
Image of Panel EC3 featuring two bison depictions, EC3.1 and EC3.2, facing right and depicted in black pigment.

**Figure 7:**
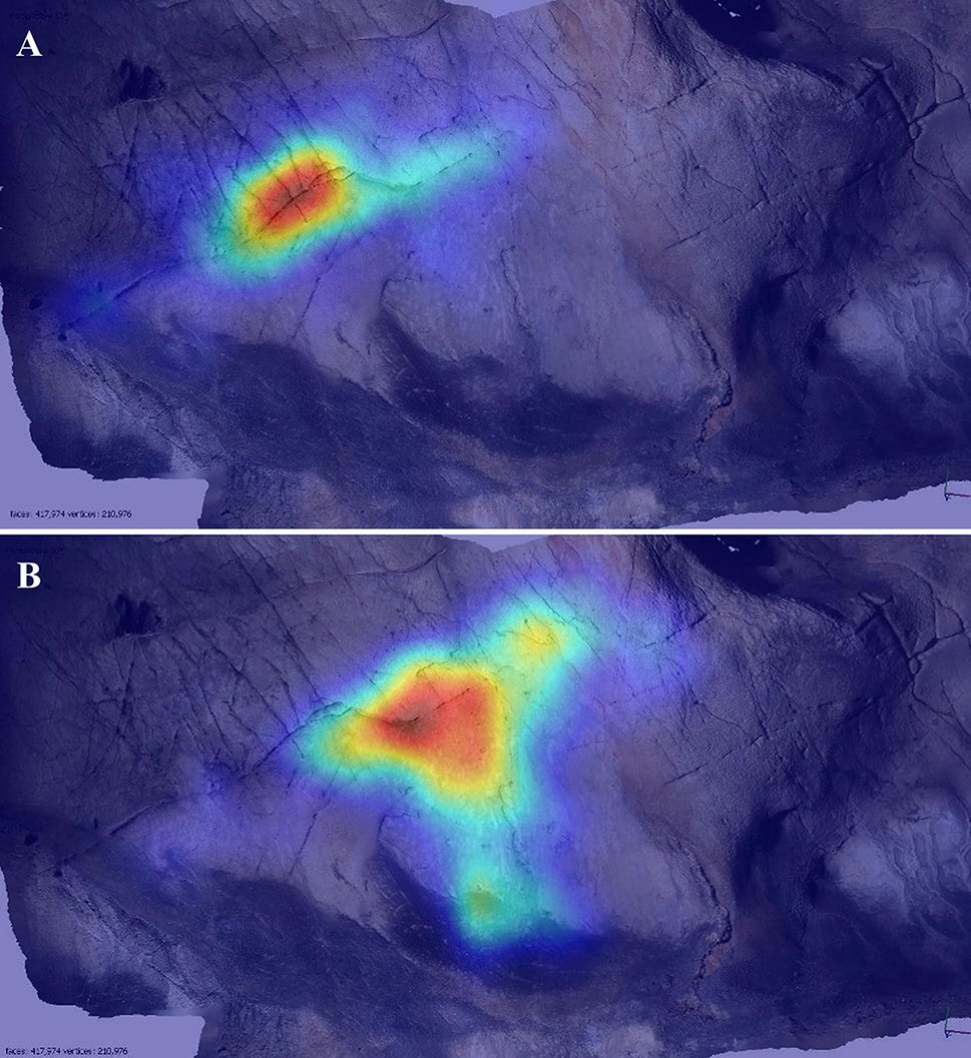
Heatmaps of tracked eye movements for participants EP14 and P10 during active observation. (**A**) EP14, corresponding to their description of depicting a horse. (**B**) P10, corresponding to their description of depicting a mammoth. This indicates that despite variation in pareidolic responses, visual attention was still focused around the same feature—a horizontal crack in the panel—that was utilised for the depiction of a bison (EC3.2).

The most pronounced responses derived from Panel EC3 (Fig. 6). 9 participants focused their attention on a horizontal crack (Fig. 7) utilised by Palaeolithic artists as the dorsal line of a large bison and the horns of a small bison, and perceived it as evocative of different animal profiles (Table 3). These identifications varied by participant among horse, bison, and mammoth. Despite this variety, all participants that expressed pareidolic responses consistently perceived the crack as evocative of an animal’s dorsal line and head: even participants that did not experience pareidolia still indicated that their depictions would be structured around the crack (e.g. using it as ground or mountains; features that do not appear in Palaeolithic art). The results thus appear to support the three hypotheses.

**Table 3:** Summary of participant responses to Panel EC3, with responses evaluated in relation to the three hypotheses.

Participant	Pareidolic response? **(H1)**	Location correct? **(H2)**	Species correct? **(H3)**	Additional comments
P1	Yes	Yes	No	Perceived horses involuntarily in natural horizontal crack used for EC3.1 and EC3.2
P2	No	No	No	Described horses, stags or deer, but did not correspond to the same area as Palaeolithic depictions
P3	Yes	Yes	No	Perceived mammoth in the crack used for EC3.2; also described ibex not relating to natural features
P4	Yes	Yes	No	Perceived crack as evoking antlers, and described drawing stag
P5	No	Yes	No	Used crack to frame scene of multiple antelopes
P6	Yes	Yes	No	Perceived crack as resembling a mountain, and depicting ibex
P7	No	Yes	Yes	Described bison using crack; did not describe crack as looking-like bison
P8	Yes	Yes	No	Perceived horse head in crack
EP9	Yes	Yes	Partially	Perceived crack as resembling mammoth; changed response to bison
P10	Yes	Yes	No	Perceived crack as resembling mammoth
P11	No	Yes	No	Described hunting scene/herd of animals across crack
P12	No	Yes	No	Used crack as horizon/landscape, with multiple animals/herds
P13	Yes	Yes	No	Perceived crack as resembling mammoth, changed response to horse dorsal line
EP14	Yes	Yes	No	Perceived crack as resembling several horse heads

### Panel EC4

**Figure 8:**
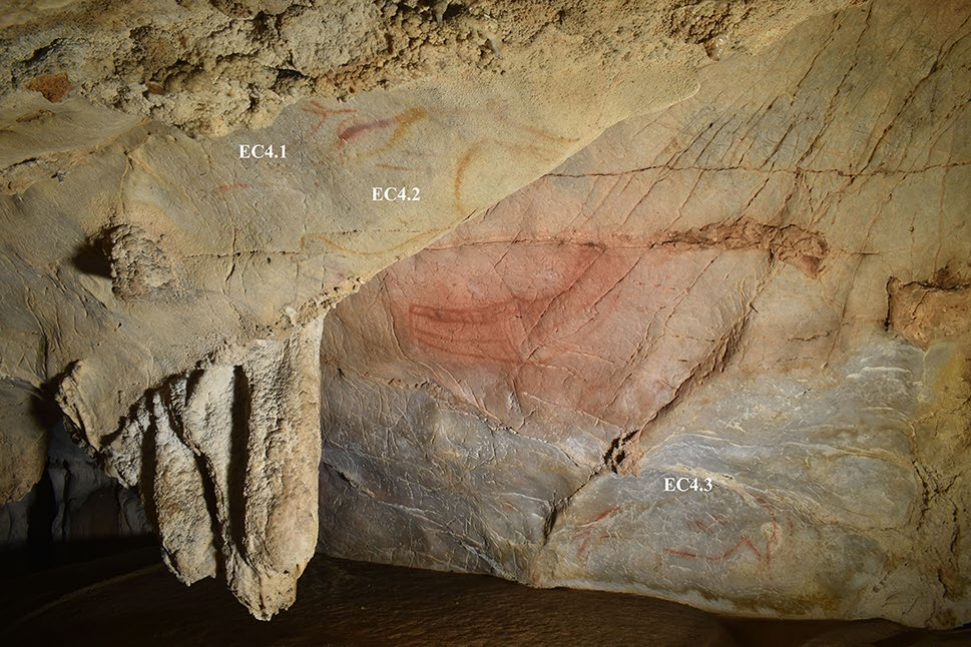
Image of Panel EC4. This panel features three animal depictions and one non-figurative “sign”. EC4.1: small partial hind in red, facing right; EC4.2: partial horse depiction in yellow, facing right, EC4.3: complete horse in red and yellow, facing left with head bent downwards.

Panel EC4 (Fig. 8) provoked the fewest pareidolic responses: only three participants perceived animals in its natural features and none of these corresponded to areas used for Palaeolithic depictions (Table 4). The topography of the panel is complex—both overhang and rear wall—hence heatmaps could not be produced appropriately on a flat 2D image. However, participants’ audio responses synchronised to screen-captured videos of the VR session did indicate that attention was drawn to particular areas.

**Table 4:** Summary of participant responses to Panel EC4, with responses evaluated in relation to the three hypotheses.

Participant	Pareidolic response? **(H1)**	Location correct? **(H2)**	Species correct? **(H3)**	Additional comments
P1	No	Yes	No	No specific species, but described using the overhang and lower area of rear wall, corresponding to the areas used for Palaeolithic depictions
P2	No	Yes	No	Used lower area of rear wall and overhang to depict scene of mammoths
P3	Yes	No	No	Perceived elk/reindeer on the wall, but not corresponding to areas used for Palaeolithic art. Also described small animals on the overhang
P4	No	Yes	Yes	No pareidolic response, but described drawing a horse on rear wall and hind on overhang (same area as EC4.1 and EC4.2)
P5	No	Partially	No	Used overhang to depict female deer (same area as EC4.1 and EC4.2), and different area of the rear wall to depict reindeer
P6	No	No	No	Described mammoth on rear wall, towards left
P7	No	No	No	Describes hunting scene across rear wall
P8	Yes	No	Yes	Perceived horse on rear wall,but not corresponding to areas used for Palaeolithic art
EP9	Yes	No	No	Perceived bison in rear wall, but not corresponding to areas used for Palaeolithic art
P10	Yes	No	No	Perceived deer in rear wall but not corresponding to areas used for Palaeolithic art
P11	No	No	No	Described multiple animals as hunting scene across rear wall
P13	Yes	No	No	Perceived ibex, lion’s head and otter on rear wall, but not corresponding to areas used for Palaeolithic art
EP14	No	No	No	Described generic animals, not corresponding to areas used for Palaeolithic art

Note that participant P12 had an incomplete session, and thus there are no results from P12 for this panel.

Several participants described the panel’s overhang feature as suitable for depicting animals, but primarily referred to both its spatial position and relatively untextured surface as the reason for its suitability. Although 4 participants noted they would produce small depictions of animals here, this often did not correspond to a perception of animal forms in natural features of the surface. Rather, it was the spatial area that constrained the depictions produced and incidentally resulted in some participants describing the depiction of similar motifs as the Palaeolithic art (i.e. female deer). For the panel’s rear, most participants appeared to focus on the undulating upper-right surface. Some expressed pareidolic responses to the features here, but these were highly varied between participants, with no two describing either the same animal or focusing on the same feature—and none of these corresponded to the areas used for Palaeolithic depictions. Other participants noted their difficulty responding to the question “what would you depict on the cave wall?”; some answered vaguely and others decided that the panel was not suitable. This suggests that depictions on this panel were not necessarily motivated by pareidolia, and that the pareidolic responses to other panels had aided participants in structuring their depictions.

## Discussion and conclusion

Our interdisciplinary VR research has enabled us to establish, for the first time, that modern participants do have pareidolic responses to cave walls under immersive simulated conditions. Although we emphasise the preliminary and qualitative nature of our results, they hint at the varying extent to which pareidolia motivated the production of Palaeolithic depictions for the four panels evaluated in El Castillo cave. Two panels, EC1 and EC3, demonstrate that those features of the cave wall that were salient to modern participants corresponded to the same areas that were salient to Palaeolithic artists. In these two panels, participants appeared to have experienced pareidolia in response to these features, describing how they would depict animals in response to the pareidolic imagery they perceived. For some, this imagery corresponded to the same animals depicted by Palaeolithic artists—for example, P3 perceiving a bison depiction on the lower, central area of Panel EC1 in the same place as the bison depiction EC1.5 was drawn.

This lends pilot independent support for the notion that pareidolia acted as a fundamental mechanism that drove the production of art in caves during the Palaeolithic. It would be premature to conclude that ‘cave art’ was actually *caused* by the perception of animals in the topographies of cave walls, but the artists of this period had intimate familiarity with the prey animals they depicted, with an acute ability to process the fragmented forms of these animals hiding behind vegetation, or rapidly identify salient forms of animals in the distance^28,66,67^. It is logical to hypothesise that the overwhelming importance of such animals to their survival primed their visual system to perceive animal forms in evocative contours; just as modern people resolve a breadth of different visual stimuli as resembling faces of people or outlines of pets^68^. Whilst our results demonstrate that immersive cave-like conditions elicit pareidolic responses to topographic features in modern participants, pareidolia may have influenced the production of other art forms. Upper Palaeolithic portable art also integrates natural features of bones and stones and this may also reflect pareidolic responses, whether or not under evocative lighting conditions^56^. VR experimental approaches to portable art objects should be critical to developing deeper insights into the extent and nature of pareidolic stimulation of the emergence and dominance of animal representations in the Upper Palaeolithic. A larger, data-rich and cross-culturally aware project should address whether these assumptions hold.

We acknowledge that these limited results are preliminary, serving primarily to demonstrate that carefully constructed VR experiments using visual psychological research methods should enable robust hypothesis testing. Larger samples, particularly incorporating cross-cultural participants that should control for ‘western bias’, combined with larger art databases and more specific questions should indeed allow us to investigate the extent and specificity of pareidolic effects on early art. Our pilot results thus encourage more nuanced investigations of the way pareidolia may (or may not) manifest in the production of Palaeolithic art, and of the conditions conducive to triggering this response. The results from Panel EC2 which do not appear to support any of the hypotheses are pertinent here. Participants’ visual attention to this panel generally focused on the lower, amorphous area of the wall which may represent a Western preference for a “blank canvas” in art production. As such, their responses were not motivated by perceiving evocative animal forms, but by cultural preferences for particular surfaces. We are encouraged by this to build in cultural variation to our participant base in future elaboration of the methodology, e.g. national, ethnic, occupational biases. Do these results suggest that different artists had different susceptibilities to pareidolia? This could be investigated by evaluating, for example, stylistic differences between images and other features (i.e., skill) which might imply distinct artists. The role of these facets have been extensively discussed in archaeological research^69,70^, alongside considerations of how different contextual features (e.g., acoustics, tactility, darkness, spatial placement)^71–75^ may have affected art making in caves, and thus our results must be contextualised with sensitivity to other mechanisms involved in Palaeolithic art production. It is important to stress that we cannot generalise about the role of pareidolia for all examples of Palaeolithic cave art. As demonstrated by our VR results, pareidolia may have had a stronger or weaker effect depending on the specific context of making, and this nuance must be appreciated^58^.

The development and piloting of an interdisciplinary VR experiment has enabled us to test specific hypotheses relating to the role of pareidolia in Palaeolithic art, and generated nuanced insights into the *extent* to which this perceptual response influenced the form and placement of depictions. Whilst it has been long suggested that pareidolia played some role in Palaeolithic art making^28–31,58^, specific hypotheses have never previously been tested, partly due to the limitations of the archaeological record. VR, and an interdisciplinary collaboration between archaeology and visual psychology, offered one tangible solution to this fundamental issue, and we argue that interdisciplinary use of VR experiments holds significant potential for providing a more nuanced understanding of the interaction of the artist, surface, and the role of visual psychological responses in the production of early art. We stress that this is a pilot study perhaps best seen as demonstrating proof of the concept that, in principle, VR-situated visual psychological research can be used to test hypotheses regarding early art making, particularly where archaeological evidence alone is insufficient. It is limited to the European Late Upper Palaeolithic, and while the specifics of our preliminary results need not hold more widely across time and space we hope that the methodological efficacy we’ve shown should pave the way for powerful interdisciplinary research into the emergence and early evolution of human visual culture.

## Materials and methods

### Ethical statement

The study was ethically approved by the Department of Archaeology, Durham University ethics sub-committee. All guidelines were followed according to this ethical approval. All participants provided informed consent for their participation in the study and were informed that they could remove their consent at any time during or after the study. As VR can be disorienting and has been known to induce feelings of motion sickness, participants were also instructed that they could pause or stop the study at any point if they felt uneasy with using VR. All data collected from participants was anonymised, and no personal information was collected from the participants.

### Participants

Fourteen participants were recruited in total for the study (biological sex: M = 8, F = 6). Twelve of these participants indicated they had no or very limited knowledge of Palaeolithic cave art, and were recruited through an open call to undergraduate and postgraduate students in the Department of Archaeology, Durham University. Two participants were Palaeolithic art experts, identified by their current active research within the field, and were recruited through direct invitations. The Palaeolithic art experts had no previous knowledge of the study or its aims, had not conducted research in or visited El Castillo cave, nor had previously seen materials directly relating to the study.

### Priming

Participants were first primed to identify Pleistocene animals, selected on the basis of available faunal assemblages from the Upper Palaeolithic levels from El Castillo^76,77^. The animals included were: horse; red deer; reindeer; ibex; chamois; mammoth; megaloceros. Participants were presented with images of the animals that became gradually more difficult to visually discriminate. This was intended to mitigate against participants’ lack of familiarity with Pleistocene animals and encourage participants to visually focus on the salient features of the animals. Participants were given feedback after each round, to encourage perceptual learning of animal profiles.

### Virtual reality

Four VR cave environments were constructed for the psychology experiments in the gaming software, Unity, to be compatible with an HTC Vive headset and controllers. Four 3D photogrammetry models of cave art panels from El Castillo (Cantabria, Spain) were integrated into four different constructed cave environments, alongside assets such as boulders and speleothems to create an immersive, naturalistic virtual environment. The 3D models had modified .jpeg textures that had the Palaeolithic art removed, preserving the natural colouration and texture of the cave wall. The virtual environment was designed to encourage active engagement with participants needing to navigate to the target wall, occasionally moving around boulders or speleothems; active engagement is important for heightening immersion in VR and thus encourages naturalistic responses^51^, especially when participants' own bodily movements are represented^40^. Participants navigated to the target wall using the joystick, but were then able to freely physically move around the target wall, actively interacting and adjusting bodily positions (e.g., crouching to look at lower areas of the target wall). Each virtual cave was approximately 40 m x 30 m, and had ambient lighting set to an intensity of 0.2 to create near absolute dark conditions simulating the conditions experienced by Palaeolithic people; all panels selected would have been beyond the reach of natural daylight in the cave.

Participants were provided with a virtual torch, held in one of their hands and visible in VR, which allowed them to illuminate the photogrammetry models and have full control over manipulating the lighting of these models and likely enhanced their sense of embodiment in VR. The virtual torch simulated the characteristics of a Palaeolithic lamp or torch. It was scaled to cast a flickering light over a radius of 2 m (diameter of 4 m) consistent with estimates and experimental observations for the light cast from torch technologies available in the Upper Palaeolithic^10,64,65^. The intensity was set at an arbitrary Unity value of 1. The intensity values for light in Unity have no real-world comparison, but an intensity of 1 provides a moderate amount of light that appears visually comparable to the light expected from a small flame. The colour of the light was set to neutral-warm of a colour temperature around 2100 K. This is a slightly cooler tone than the light recorded for experimental torches^65^, but enabled the participants to have more clarity within the VR environments than a warmer toned light and was selected to reduce disorientation for the participants. Small virtual hearths which cast a warm light over a radius of 2 m were placed either side of the target wall(s) to help guide the participant. These hearths were placed to ensure the light did not illuminate the photogrammetry model, so that the models were only illuminated by the virtual torch held by the participant.

### Eye tracking

Eye tracking was used to determine which natural features drew the visual attention of the participants, both identifying the features which were cumulatively paid the most visual attention by a participant and the saccade of visual attention across a wall. This also allowed for a comparison between both the idle and active observation of a participant, and between participants that had strong pareidolic responses to a wall and those that had no pareidolic response to the same wall. To achieve this, an additional calibration routine was implemented in C# within Unity (available upon reasonable request). This initially measured the centred direction of gaze at multiple head orientations. These calibrations were then used to reduce slippage errors in the in-built HTC Vive headset eye tracking system. Gaze direction estimates were continuously corrected using participants’ current head orientation to interpolate between the nearest three head orientation calibrations. This resulted in a reduction of gaze estimate error (better than + / − 2 degrees) sufficient to determine whether gaze followed features in cave walls. The tracked eye movements were recorded through a screen captured video of the participant’s view in VR. Tracked eye movements were recorded at a rate of 30 times per second, corresponding to the frame rate of the screen captured video.

The innovative use of eye tracking in VR within this research meant there was no pre-established method for processing the data. As the VR experiments allowed participants to experience 6 degrees of freedom (6-DoF, i.e. participants were able to rotate their head, rotate and move their body, and navigate through the VR space) to increase immersion, this caused some degree of difficulty for processing the tracked eye movements. Although VR has been increasingly used in psychological research^51^, eye tracking in VR is a relatively new technological development^78^ and has only recently been pioneered in visual psychology studies^49^. Visual psychological studies tend to restrict participants to viewing an image in virtual reality with only 3-DoF i.e., participants’ movements were limited to only moving and rotating their head^47,48,50^. This enables high levels of accuracy and ease in processing eye movements for these studies, but reduces immersion for the participants, limiting their ability to actively engage with the VR environment. Whilst this is undoubtedly beneficial for certain kinds of research in visual psychology, the lack of participant immersion would have been a significant limitation for this research. An archaeological study has recently used eye tracking in VR with 6-DoF by using ArchGIS Pro to process areas of visual attention indicated by gaze direction within a Unity VR environment^58^. This provides tracked eye movements for general areas of visual attention (i.e. a particular wall or object), but perhaps is limited in capturing the higher level of accuracy in visual attention required for understanding the specific features of a cave wall (e.g. a small crack or fissure) that may be drawing visual attention. Some creative, albeit complex, solutions for tracking eye movements to this level of detail in a VR environment with 6-DoF do exist^49^, but there is currently no standard for processing this kind of data.

Tracked eye movements were thus visualised by a small dot within a screen-captured video of the participant’s VR experience. Visualising the tracked eye movements in this way enabled participants to correct the eye tracking if they felt it did not represent their eye movements, by performing an additional calibration at any point during the experiment. The tracked eye positions were recorded through a screen-captured video of the participant’s view in VR, and the visualised eye positions were analysed frame-by-frame to manually plot the eye movements onto a 2D image of a particular panel’s 3D model. The 2D image aimed to capture a neutral perspective i.e., from the perspective of a participant looking at the wall from an upright, central position. The plotted eye movements were processed through MATLAB to create a heatmap of the areas which were cumulatively paid the most visual attention by a participant. Automating this process was not possible; as the participants experienced the environment with 6-DoF, any automated program would have to recognise the same point from multiple different angles and distances which would have necessitated a lengthy process of machine learning. Due to the high frame rate of recorded eye movements (30 times per second), the time intervals selected for plotting eye movements was restricted to between 30 and 60 s. Although this resulted in figures which provided only a snapshot of the eye movements of a participant observing a wall, it enabled each eye tracking figure to correspond to a specific response of a participant or the idle observation period before the participant was asked questions. Invariably, the process of manually plotting tracked eye movements resulted in some limitations to the data analysis. Heatmaps were only able to be produced for three out of the four cave art panels. For panel EC4, its complex morphology caused difficulty in creating 2D heatmaps; participants explored all three dimensions of this cave wall, which meant eye movements could not be plotted onto a 2D neutral image of the cave wall.

## Data Availability

The raw dataset is available upon reasonable request to the corresponding author.
